# Supplementary material for: Human Pluripotent Stem Cell-Derived Alveolar Organoid with Macrophages
Source: Int J Mol Sci. 2022 Aug 16;23(16):9211. doi: 10.3390/ijms23169211 (PMC9409017; doi:10.3390/ijms23169211)
Supplement: Supplementary file 1 [file ijms-23-09211-s001.zip › Supplemental information.pdf]

Supplemental information

Human pluripotent stem cell derived alveolar organoid with macrophages derived from human pluripotent stem cell

Ha-Rim Seo[1ë], Hyung-Joon Han[2], Young-Woock Noh[1], Youngson Lee[2], Seung-Ju Cho[1*], Jung-Hyun Kim[2*],

[1]Division of Drug Evaluation, New Drug Development Center, Osong Medical Innovation Foundation, Cheongju 28160, Republic of Korea
[2]Division of Intractable Diseases, Center for Biomedical Sciences, Korea National Institute of Health, Korea Centers for Disease Control and Prevention, Cheongju 28159, Republic of Korea

[*]These authors are corresponding authors.

Video S1. Production of 3D lung organoid from CD47hiCD26lo lung progenitor cells. (A) The time lapsed image of growing of 3D lung organoid from CD47hiCD26lo lung progenitor cells. The time covers a period of about 48 hours, with a 2 hours time interval between images. Recordings and data processing were made with a IncuCyte system using a 4X objective lens. Scale bar = 800 μm.

Video S2. Macrophage induction into lung organoid. (A) The live image of injection of macrophage into lung organoid. The time covers a period of about 10 seconds, with a whole live images. Recordings and data processing were made with a Ocam system using a 10X objective lens. Scale bar = 200 μm.

Supplemental Figure S1. Macrophage induction into the lung organoid in time dependent manner. (A) Representative phase contrasts and fluorescent dye images of macrophage-included lung organoid from hESCs at induction day 1 to 14. Scale bar = 100 μm. (B) Representative phase contrasts and fluorescent dye images of macrophage-included lung organoid from hIPSCs at injection day 1 to 14. Scale bar = 100 μm.
