# Supplementary figures and images for: Human Pluripotent Stem Cell-Derived Alveolar Organoid with Macrophages
Source: Int J Mol Sci. 2022 Aug 16;23(16):9211. doi: 10.3390/ijms23169211 (PMC9409017; doi:10.3390/ijms23169211)

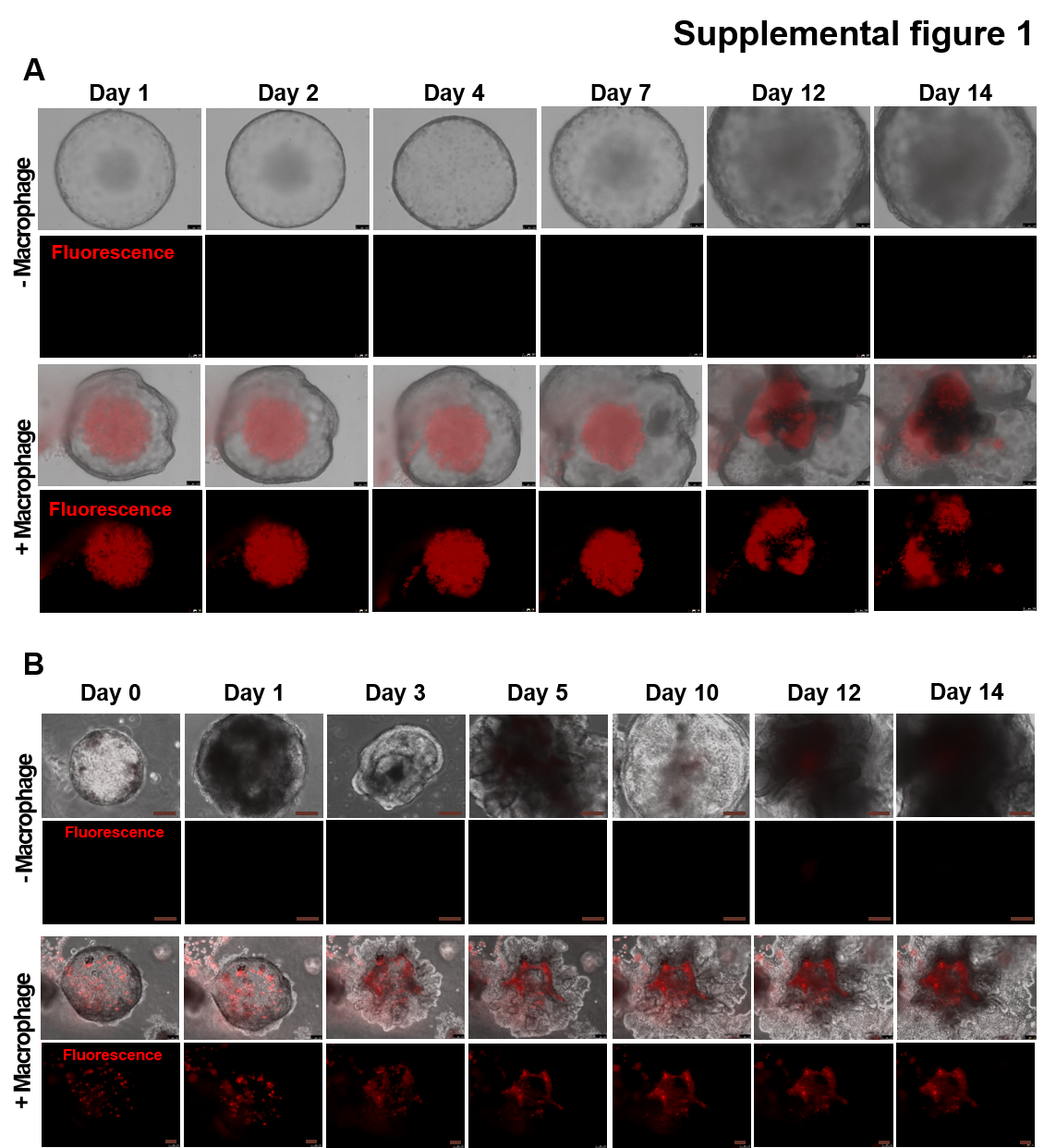

Supplement: Supplementary file 1 [file ijms-23-09211-s001.zip › Supplemental Figure S1.tif]
